# Assessment of functional and cosmetic outcomes of Extended-Tubularized incised plate for mid-shaft and distal hypospadias as a modification of Snodgrass repair: a cross-sectional study

**DOI:** 10.1097/MS9.0000000000001136

**Published:** 2023-08-07

**Authors:** Hayder H. Alwaeli, Hasanain F. Hasan Al-Timimi, Mohammed B. Ismail

**Affiliations:** a Department of Surgery, Al-Kindy Teaching Hospital; b Department of Surgery, College of Medicine, University of Baghdad, Baghdad, Iraq

**Keywords:** hypospadias, outcome, Snodgrass repair, tubularized incised plate

## Abstract

**Background:**

Hypospadias is a congenital abnormality of anterior urethral and penile development where the urethral meatus is ectopically located on the ventral aspect of the penis. It is a relatively common condition affecting ~1 in 250 male birth. Extended-Tubularized Incised Plate (E-TIP) is a modification of Snodgrass repair by extending the midline incision to the apical part of the glans resulting in a more normal appearing location of the meatus and straight urinary stream without increasing the risk of complications.

**Aim of study:**

To evaluate functional and cosmetic outcomes of E-TIP repair for mid-shaft and distal hypospadias as a modification of Snodgrass repair.

**Patient and method:**

A prospective cross-sectional study including 53 cases of mid-to-distal hypospadias repaired with the E-TIP technique between November 2019 and February 2022 in Baghdad. The authors start with the standard technique described by Snodgrass, but the midline incision of the urethral plate extended up to the apical part of the glans tip, and tabularization started distally creating a slit-like a neomeatus. Outcome assessment depended on an objective evaluation of the following parameters: maximum flow rate, post-void residual, Hypospadias Objective Score Evaluation (HOSE) score, and direction of the urinary stream.

**Results:**

The mean age was 3.4±2.1 years, ranging from 0.6 to 9 years. The mean duration of follow-up was 14.4±6.9 months, and the location of the meatus was coronal in 16 patients (30%), subcoronal in 25 (47%), and mid-distal shaft in 12 (23%). The glans width was greater than or equal to 14 mm in 46 patients (87%) and less than 14 mm in 7 (13%). Q.max was above the fifth percentile of age-related uroflowmetry nomograms for healthy children 5–15 years old in 29 boys (93.5%) and below the fifth percentile in 2 (6.5%). Post-void residual urine was less than 10% of voided volume in 28 out of 31 boys (90%) and greater than 10% in 3 (10%). Regarding cosmetic outcomes, 50 boys (94%) had good penile appearance and their HOSE score greater than or equal to 14 with a slit-like meatus located at the tip of the glans, while 3 (6%) of them their score was less than 14. The overall mean HOSE score postoperatively for all patients was 15±0.9. Six patients (11%) had a deviated urinary stream and 47 (89%) had a straight stream.

**Conclusion:**

E-TIP repair is a good alternative to standard Snodgrass repair for mid-shaft to distal hypospadias with a good functional and cosmetic outcome, particularly the neomeatus position at the tip of the glans, which is similar to the normal one without increasing the risk of meatal stenosis.

## Introduction

Highlights

- Hypospadias is a congenital abnormality of anterior urethral and penile development.
- It is relatively a common condition.
- We evaluated the functional and cosmetic outcomes of Extended-Tubularized Incised Plate as modification of Snodgrass repair.

Hypospadias is a congenital abnormality of anterior urethral and penile development. The urethral meatus is ectopically located on the ventral aspect of the penis. In more severe forms, the urethral opening may be located at the scrotum or perineum and may be associated with the chordee or bifid scrotum^1^.

Hypospadias is a relatively common condition affecting ~1 of 250 male births, and its incidence increases in boys born prematurely, and small for gestational age, and with low birth weight^2,3^.

The primary treatment of hypospadias is surgical correction; more than 400 different types of repairs that have been described in the medical literature^4^.

A successful surgical repair was defined as a straight erection and the meatus near the tip of the glans, allowing voiding in a standing position and smooth sexual intercourse^5^.

Tubularized incised plate (TIP) was introduced by Snodgrass in 1994, and nowadays it is the most commonly used technique for surgical repair of mid-shaft and distal hypospadias. It relies on an incision in the urethral plate to allow tension-free tubularization^6^.

According to the principal technique described by Snodgrass, it is better not to incise the distal part of the glans to reduce the risk of meatal stenosis; this resulted in a higher rate of achieving an oval, slit-like meatus but reducing the chance of locating the meatus in the glans tip^7^.

In this study, we tried a modification of Snodgrass repair by extending the midline incision to the apical part of the glans, resulting in a more normal appearing location of the meatus and a straight urinary stream without increasing the risk of meatal stenosis. This modification was previously proposed by Jayanthi^8^. The functional and cosmetic results of this modification were objectively assessed with a uroflowmetry study and by the Hypospadias Objective Score Evaluation (HOSE) score (Table 1)^9^.

**Table 1:** HOSE score^9^.

Variable	Description	Score
Meatal location	Distal glanular	4
	Proximal glanular	3
	Coronal	2
	Penile shaft	1
Meatal shape	Vertical slit	2
	Circular	1
Urinary stream	Single	2
	Spray	1
Erection	Straight	4
	Mild angulation <10°	3
	Moderate>10° but <45°	2
	Severe>45°	1
Fistula	None	4
	Single subcoronal	3
	Single proximal	2
	Multiple or complex	1
Total score		16

## Patients and methods

A prospective cross-sectional study including 53 cases of distal hypospadias repaired with extended TIP repair (E-TIP), a modification of Snodgrass repair, between November 2019 and February 2022.

Ethical considerations were obtained according to the Helsinki Declaration. Informed written consent was obtained after explaining the nature of the operation and its risks. The approval of the ethics committee was obtained from the Health Ethics Committee in College of Medicine, University of Baghdad, with a registration number: 40. The methods in this article were prepared according to strengthening the reporting of cohort, cross-sectional, and case–control studies in surgery (STROCSS) criteria^10^. The research was registered at researchregistry: https://www.researchregistry.com with a unique identifying number researchregistry8992.

Inclusion criteria were if the location of the meatus was coronal, subcoronal, and mid-distal shaft; age less than 10 years; exclusion criteria including proximal hypospadias, previous failed surgery, megameatus, glandular hypospadias, and age more than 10 years.

The study sample size was determined by the Raosoft sample size calculator, considering a 95% CI and a 7% margin of error.

### Surgical technique

After counseling and taking informed consent from the patient’s caregivers, under general anesthesia in a supine position after painting and draping, then documenting the meatal location by measuring the urethral plate width, length, transverse glans diameter, and degree of curvature if present (after degloving). We start with the standard technique described by Snodgrass^6,7^; then the midline incision of the urethral plate extended up to the apical part of the glans tip and tabularization started distally creating a slit-like neomeatus at least 3 mm and continuing proximally with two layers repair using 6/0 Vicryl suture. A feeding tube (6–8 F) were used as a stent and usually remain for 1 week; the neourethra was covered by a double dartos layer then completing glanuloplasty, circumcision, skin suturing, and dressing. The mean operative time was 93.2±11.7 min.

The patient is usually discharged home on the same day on oral antibiotics until stent removal; the dressing is exchanged on the fifth postoperative day, and the stent is removed at the 7th day. The patient is usually reviewed at the time of stent removal and then two times within the first month, at 3 months and 6 months. At each visit, we examined the penis, including the shape and location of the meatus urinary stream and the presence of complications (infection, meatal stenosis, glans dehiscence, fistula, etc.).

### Evaluation of the outcome

In this study, we depend on objective functional evaluation at a 3-month visit, considering the following parameters: *Maximum flow rate*: (in toilet-trained boys and if voided volume>50 ml). A maximum flow rate (Q.max) below the fifth percentile range utilizing an age-related uroflowmetry nomogram for healthy children 5–15 years old^11^ is considered abnormal. *Post-void residual (PVR):* measured by bedside ultrasonography considered abnormal if it was greater than 10% of voided volume. *HOSE (Hypospadias Objective Scoring Evaluation) score*: to evaluate the cosmetic aspect of the outcome, it is calculated by another urologist who was not involved in surgery; the scoring ranges from the worse score (5) to the best score (16) that presents the highest score of each variable as shown in Table 1. A score of greater than or equal to 14 is considered an acceptable cosmetic outcome. *Direction of urinary stream*: if it is straight of deviated.

The patients’ data were saved in an Excel program. The results were arranged in tables and figures, as numbers and percentages, and a statistician reviewed them.

## Results

Fifty-three boys were included in this study; their mean age was 3.4±2.1 years ranging from 0.6 to 9 years. Four patients missed follow-up after few months, the mean duration of follow-up of the remaining (49) boys was 14.4±6.9 months.

**Figure 1:**
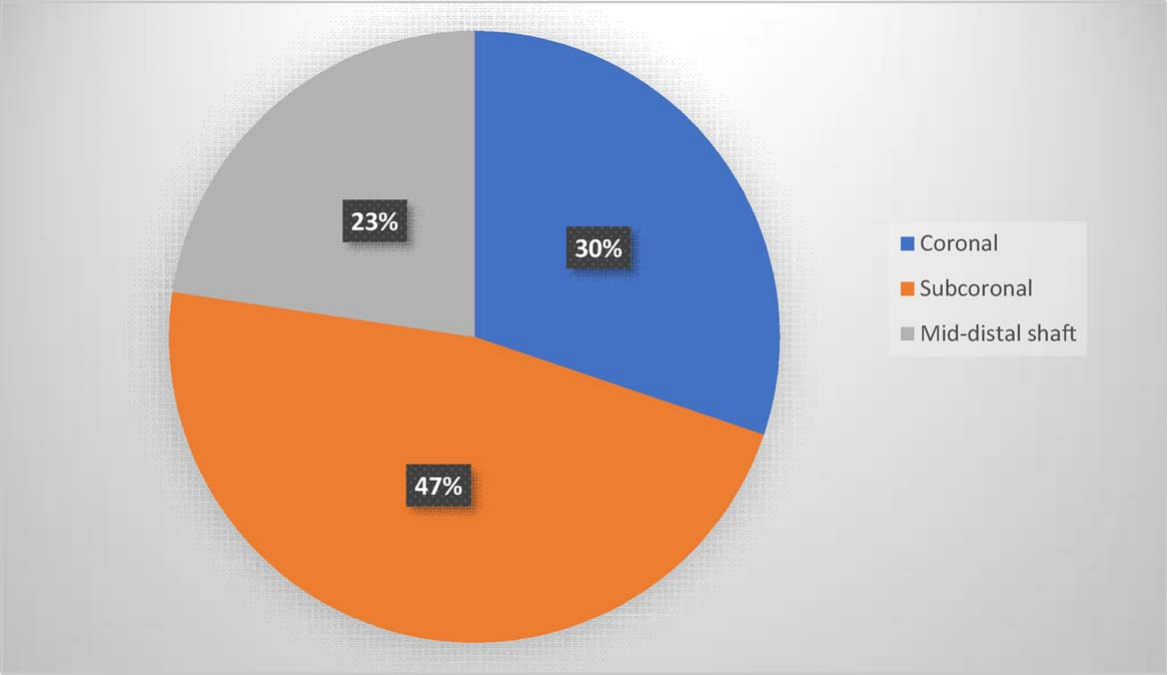
Distribution of meatal location among patients.

The location of the meatus was coronal in 16 patients (30%), subcoronal in 25 (47%), and mid-distal shaft in 12 (23%) (Fig. 1).

**Figure 2:**
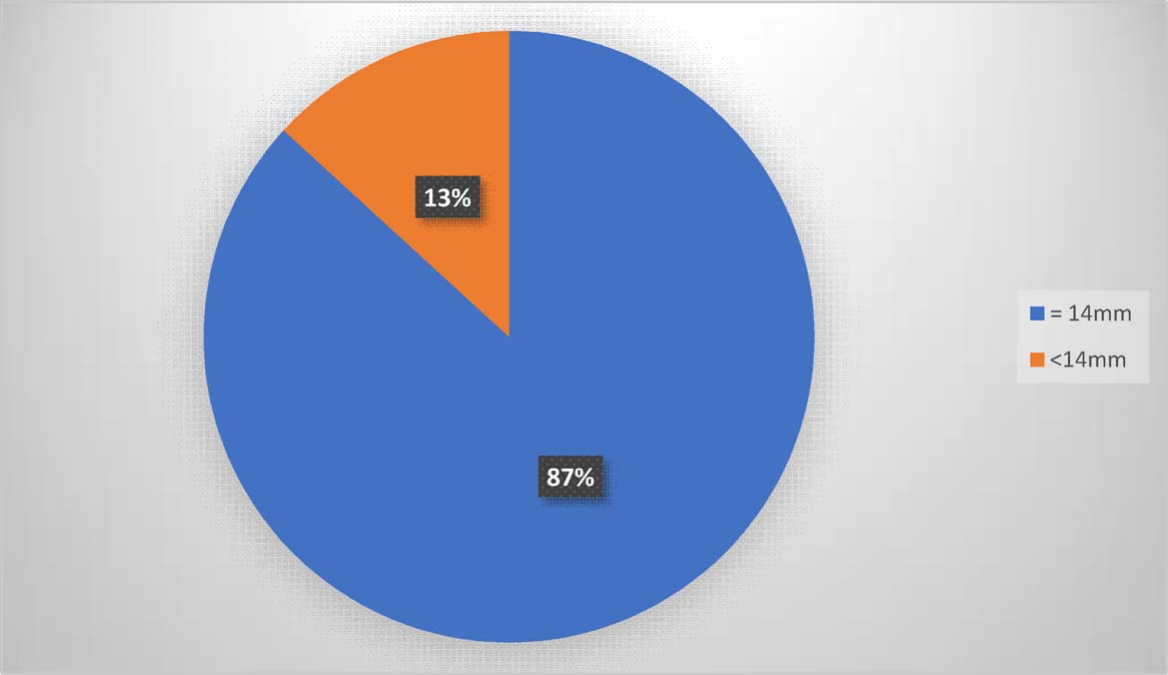
Distribution of glans width among patients.

The glans width was greater than or equal to 14 mm in 46 patients (87%) and less than 14 mm in 7 patients (13%) (Fig. 2).

**Figure 3:**
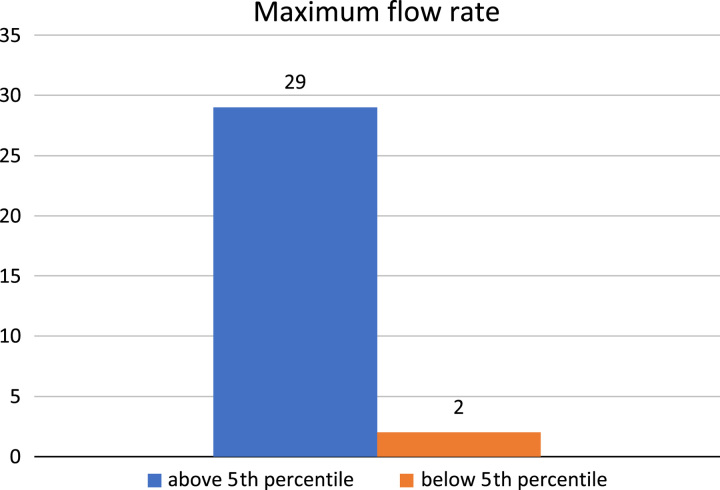
Postoperative maximum flow rate utilizing age-related uroflowmetry nomograms for healthy children 5–15 years old^11^.

Uroflowmetry and post-void volume were obtained between 3 and 6 months postoperatively in boys who were toilet-trained (31; 58.5%); Q.max was above the fifth percentile of age-related uroflowmetry nomograms for healthy children 5–15 years old in 29 boys (93.5%) and below the fifth percentile in 2 (6.5%); one of whom had meatal stenosis and was subjected to a meatotomy later (Fig. 3).

**Figure 4:**
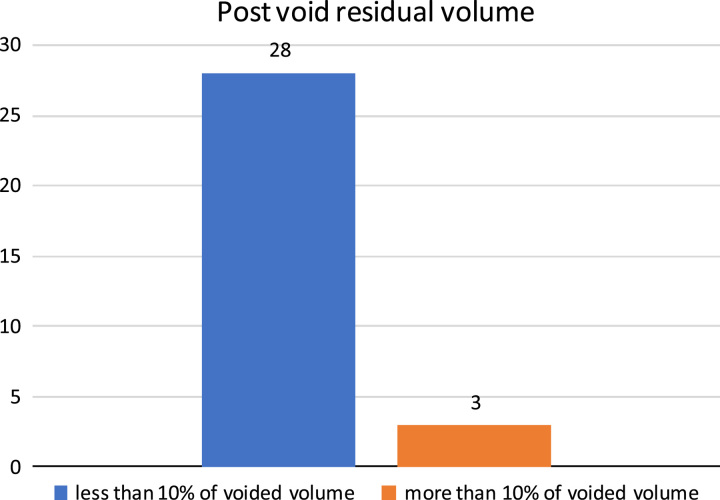
Postoperative post-void residual volume.

PVR urine was less than 10% of voided volume in 28 out of 31 boys (90%) and greater than 10% in 3 (10%), one of them for the same patient with meatal stenosis (Fig. 4).

**Figure 5:**
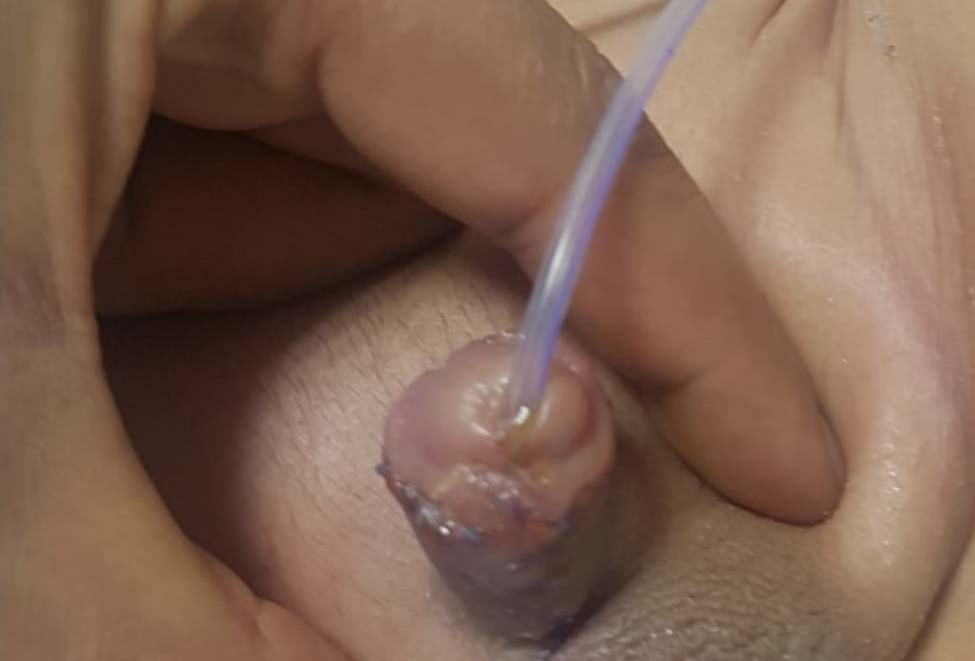
Location of meatus at the tip of the glans on seventh postoperative day.

**Figure 6:**
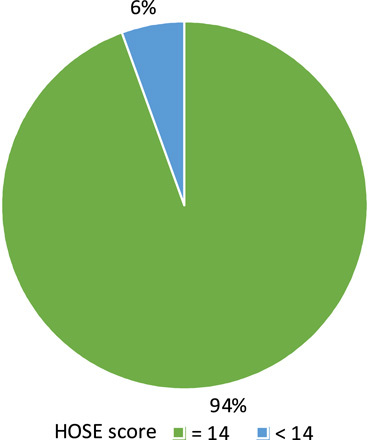
Distribution of patients postoperatively according to their HOSE score. HOSE, hypospadias objective score evaluation.

Regarding cosmetic outcome, 50 boys (94%) had good penile appearance and their HOSE score greater than or equal to 14 with a slit-like meatus located at the tip of the glans (Fig. 5), while 3 (6%) of them their score was less than 14. The overall mean HOSE score postoperatively for all patients was 15±0.9 (Fig. 6).

**Figure 7:**
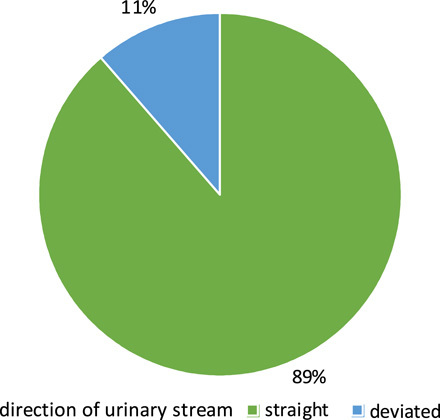
Direction of the urinary stream.

Six patients (11%) had a deviated urinary stream and 47 (89%) had a straight stream (Fig. 7).

The total complications occurred in three boys (5%); one meatal stenosis, one urethrocutaneous fistula, and one glans dehiscence due to infection.

## Discussion

Snodgrass or TIP technique is a widely accepted and preferred method for correction of mid-shaft to distal hypospadias by most hypospadias surgeons^6^. In this study, a modification of this technique that was proposed by Jayanthi^8^, which is E-TIP repair, where the relaxing midline incision is extended to the glans tip, creating a slit-like meatus at a position similar to a normal one with a straighter urinary stream without increasing the risk of complications.

In Jayanthi’s study, 110 boys underwent repair with the E-TIP technique; their mean age was 9.5 months (range 5–60), which was lower than the mean age of this study (3.4 years) ranging from 0.6 to 9 years. He concluded that no child had developed meatal stenosis^8^, while in this study, only one child developed meatal stenosis (less than 2%).

The Q.max of 29 boys (93.5%) out of 31 who were eligible for uroflowmetry test was above the fifth percentile of uroflowmetry nomograms for healthy children 5–15 years^11^, while it was below the fifth percentile only in 2 (6.5%). These results were slightly better than a similar study by Al-Adl AM *et al*. where 4 out of 26 (15%) toilet-trained boys showed an abnormal Q.max below the fifth percentile while others above the fifth percentile for the same nomogram. The PVR volume of the same study was greater than 10% of the voided volume in three boys (11%)^12^. This was comparable to the present study, where PVR was greater than 10% in 3 (10%) out of 31.

Regarding cosmetic outcome, the mean postoperative HOSE score for all 53 boys was 15±0.9, which is comparable if not better than standard TIP repair as a study by Güner and Arikan^13^ when the mean HOSE score in 35 patients who had standard TIP repair was 14.9±1.4 . Regarding the distribution of postoperative HOSE scores among our patients, 50 of them (94%) had good penile appearance with HOSE score greater than or equal to 14; however, the urinary stream was deviated in 6 patients (11%) while 47 (89%) had a straight stream. These results were comparable to that of Al-Adl *et al*.^12^ that revealed 42 of 43 boys (98%) their HOSE score greater than or equal to 14 and a straight urinary stream was attained in all boys except four (9%).

When we consider the complication rate of utilizing the E-TIP method in this study, the total complications occur in three boys (5%), one meatal stenosis, one urethrocutaneous fistula, and one glans dehiscence due to infection. These results were similar to or slightly better than standard TIP repair, for example, Aslam *et al*.^14^ reviewed 74 patients with distal to mid-shaft hypospadias who had undergone single-stage Snodgrass hypospadias repair (standard TIP). The overall complication rate occurred in five patients 7% including urethrocutaneous fistula, glans dehiscence, and meatal stenosis. The relatively low complication rate in this study may be attributed to the surgeons’ experience and the high number of cases done by each.

However, the limitations of the present study are the relatively small number of patients, the short follow-up, and it is not a comparative study with the standard TIP technique. Further studies are therefore required to address these factors.

## Conclusion

E-TIP repair is a good alternative to standard Snodgrass repair for mid-shaft to distal hypospadias with a good functional and cosmetic outcome, particularly the neomeatus position at the tip of the glans, which is similar to the normal one without increasing the risk of meatal stenosis.

## Ethical approval

The approval of ethics committee was obtained from Health Ethics Committee in College of Medicine, University of Baghdad, with a registration number: 40. Ethical considerations were obtained according to the Helsinki Declaration.

## Consent

Informed approval from patients was acquired before enrollment in this study.

This consent paper included the followings:

1. Study objectives.
2. Volunteer participation with no bonuses.
3. Free to withdrawal from project without effect on treatment course.
4. Confidentiality of data is fundamental.

## Sources of funding

I declared all sources of funding and declare the role of study sponsors, if any, in the collection, analysis and interpretation of data; in the writing of the manuscript; and in the decision to submit the manuscript for publication.

## Author contribution

All authors, did the study concept or design, data collection, data analysis or interpretation, and writing the paper.

## Conflicts of interest disclosure

The authors declare that they have no conflicts of interest.

## Research registration unique identifying number (UIN)

1. Name of the registry: www.researchregistry.com.
2. Unique identifying number or registration ID: 8992.
3. Hyperlink to registration: https://www.researchregistry.com/browse-the-registry#home. (Researchregistry 8992. Available at: www.researchregistry.com; https://www.researchregistry.com/browse-the-registry#home).

## Guarantor

No guarantor.

## Data availability statement

Data sharing is not applicable to this article.

## Provenance and peer review

Not commissioned, externally peer reviewed.
